# Essential Guide to Hydrogel Rheology in Extrusion 3D Printing: How to Measure It and Why It Matters?

**DOI:** 10.3390/gels9070517

**Published:** 2023-06-26

**Authors:** Helena Herrada-Manchón, Manuel Alejandro Fernández, Enrique Aguilar

**Affiliations:** 1 Fundación Idonial, Parque Científico y Tecnológico de Gijón, Avda, Jardín Botánico 1345, 33203 Gijón, Spain; alejandro.fernandez@idonial.com; 2 Centro de Innovación en Química Avanzada (ORFEO-CINQA), Instituto Universitario de Química Organometálica “Enrique Moles”, Departamento de Química Orgánica e Inorgánica, Universidad de Oviedo, C/Julián Clavería 8, 33006 Oviedo, Spain; eah@uniovi.es

**Keywords:** 3D printing, rheology, hydrogel, extrusion

## Abstract

Rheology plays a crucial role in the field of extrusion-based three-dimensional (3D) printing, particularly in the context of hydrogels. Hydrogels have gained popularity in 3D printing due to their potential applications in tissue engineering, regenerative medicine, and drug delivery. The rheological properties of the printing material have a significant impact on its behaviour throughout the 3D printing process, including its extrudability, shape retention, and response to stress and strain. Thus, understanding the rheological characteristics of hydrogels, such as shear thinning behaviour, thixotropy, viscoelasticity, and gelling mechanisms, is essential for optimising the printing process and achieving desired product quality and accuracy. This review discusses the theoretical foundations of rheology, explores different types of fluid and their properties, and discusses the essential rheological tests necessary for characterising hydrogels. The paper emphasises the importance of terminology, concepts, and the correct interpretation of results in evaluating hydrogel formulations. By presenting a detailed understanding of rheology in the context of 3D printing, this review paper aims to assist researchers, engineers, and practitioners in the field of hydrogel-based 3D printing in optimizing their printing processes and achieving desired product outcomes.

## 1. Introduction

Rheology is defined as the study of the physical principles that regulate the flow and deformation of matter subjected to forces. The practical use of rheology is found in areas such as quality control and production, chemical and mechanical engineering, industrial research and development, or materials science in numerous industries (pharmaceuticals, cosmetics, agriculture, food, ceramics, paints, etc.) where plastics, synthetic fibres, pastes, lubricants, creams, suspensions or emulsions constitute the raw material and are the object of study [1]. For this reason, it is currently accepted that rheology is an interdisciplinary science whose development is carried out not only by physicists, but also by engineers of various specialties, mathematicians, chemists, biologists, pharmacists, etc., giving rise to a wide range of practical application possibilities [2].

Among all the different 3D printing technologies, extrusion-based three-dimensional (3D) printing is one of the most common methods for freely digital fabrication due to its ease of use, low initial investment, and high variety of materials available. In this technique, 3D structures are built in a layer-by-layer mode, using sequential extrusion of materials from a nozzle, following a predesigned computer model [3,4]. In particular, extrusion-based 3D printing of hydrogels, namely cross-linked networks of flexible polymer chains containing a large amount of water as fill solvent, is a technology that is gaining popularity due to its potential applications in tissue engineering, drug delivery, and regenerative medicine [5,6,7,8]. Hydrogels are highly biocompatible materials that can mimic the properties of natural tissues, and the fact that 3D printing allows for the creation of high-resolution customised structures with precise control over their size, shape, and mechanical properties is one reason why this technology is so widely used at present.

Predictably, the study of rheology is of great importance within the extrusion-based 3D printing of hydrogels since, throughout the printing process, the material is exposed to various external forces and recovery or rest stages, which have a considerable impact on its properties and behaviour. Therefore, the rheological properties of the printing material determine how well it can be extruded through the printing nozzle and how it will behave during the printing process. The viscosity of the printing material and its shear thinning properties, for example, will determine how easily it can flow through the nozzle and will affect its ability to maintain its shape after being extruded. The elasticity of the material will also determine how it will respond to stress and strain during printing. Subsequently, different hydrogel parameters, such as shear thinning behaviour, thixotropy, viscoelasticity, and the gelling mechanism or temperature, are fundamental, and a good understanding of the rheological properties is necessary to optimise the printing process and achieve the desired quality and accuracy of the final product [3,9].

To understand the different types of existing fluid, their properties, and the tests necessary for their characterisation, familiarisation with the theoretical foundations of rheology, the different analyses to be carried out, and the correct interpretation of their results is required. Thus, the purpose of this work is to provide a complete review of terminology, concepts, and rheological tests and their applicability to 3D printing in the evaluation of the printability of different types of hydrogels.

## 2. Rheology Theoretical Basics

**Figure 1:**
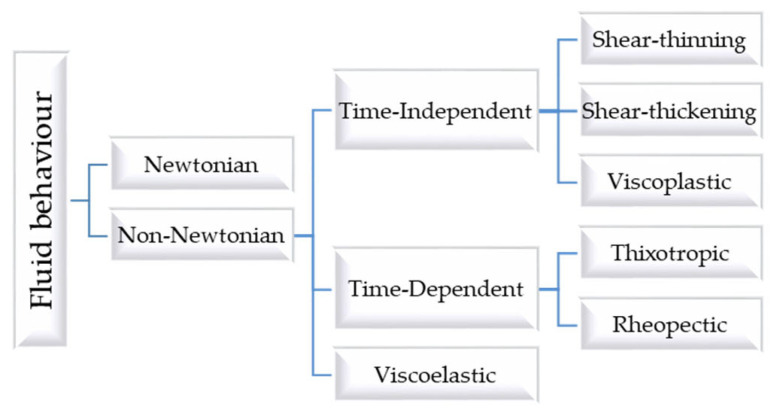
Rheological classification of fluids.

The most common classification scheme for fluids is based on their response to an externally imposed shear stress, known as rheological classification (Figure 1).

Understanding this classification requires reference to Newton’s law (1)), where τ represents shear stress (in Pa), μ represents viscosity (in Pa·s), and $\dot{\gamma }$ represents shear rate ($s^{-1}$). This shear stress or external shear force is described mathematically as the force (*F*) applied over a unit area (*A*).

(1) $$\frac{F}{A}=\tau =\mu \cdot \dot{\gamma }$$

This law indicates that for an incompressible Newtonian fluid in laminar flow, the resulting shear stress equals the product of the shear rate and the fluid’s viscosity. Thus, μ is a constant of proportionality characteristic of the material, its temperature, and pressure, and does not depend on $\dot{\gamma }$ or τ [10,11]. Viscosity reflects resistance to flow, and decreases as temperature increases and pressure decreases [12]. For a Newtonian fluid, the rheogram or flow curve, which is the graphical representation of shear stress with respect to shear rate, is a straight line with slope μ (or Newtonian viscosity) that passes through the origin. On the contrary, a non-Newtonian fluid is characterized by either a non-linear flow curve or a flow curve that does not intersect the origin. This means that the viscosity of the fluid is not constant at a given temperature and pressure and depends on flow conditions, such as flow geometry or shear rate. It can even depend on the kinematic history of the fluid element under consideration [10,13,14]. Thus, non-Newtonian fluids can be classified into three general groups:

1. Time-independent fluids, for which the shear stress at any point is determined solely by the value of the shear rate at that point.
2. Time-dependent fluids, which refer to more intricate fluid systems, where the correlation between shear stress and shear rate depends on the duration of the applied shear stress and the fluid’s kinematic history.
3. Viscoelastic fluids, which show partial elastic recovery after deformation.

It should be noted that this classification scheme is slightly arbitrary, since most real materials often exhibit a combination of two, or even three, distinct non-Newtonian behaviours. However, it is usually possible to identify the dominant non-Newtonian feature and take this as the basis for further calculations and discussions.

### 2.1. Time-Independent Fluids

In general, the behaviour of materials of this type can be described by the formula:

(2) $$\dot{\gamma }=f\left(\tau \right)$$

This formula suggests that the value of τ at any given point within the sheared fluid is solely determined by the value of the shear rate at that point, or vice versa. Depending on the shape of the function  f, these fluids can be classified into one of three categories: pseudoplastic or shear-thinning, dilatant or shear-thickening, and viscoplastic. The literature contains numerous mathematical expressions that offer empirical relationships between shear stress and shear rate or apparent viscosity, ranging in complexity and form. Some of these expressions involve direct attempts at curve fitting, while others are based on statistical mechanics [15]. While these theoretical models can be useful in silico tools for evaluating material capabilities, it is important to note that they often involve simplifications and approximations. Therefore, experimental validation is necessary to confirm the accuracy of the models in practice [9].

**Figure 2:**
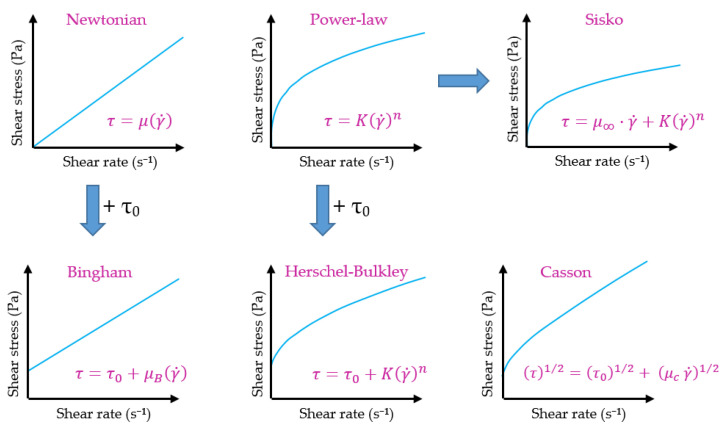
Overview of mathematical models for flow behaviour.

Time-independent fluids and the applicable mathematical models (Figure 2) are classified and detailed in the following subsections.

#### 2.1.1. Shear-Thinning or Pseudoplastic Fluids

The most common types of non-Newtonian and time-independent fluids are shear-thinning fluids. In these materials, the apparent viscosity decreases as the shear rate increases.

**Figure 3:**
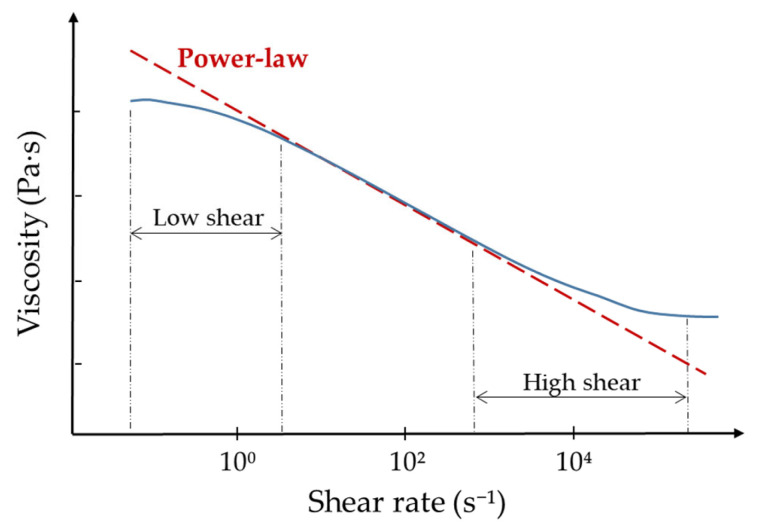
Example of application of the Power-law model (red dashed line) in an idealized flow curve (blue line).

The Ostwald-de Waele, or Power-law model, is the most commonly used mathematical model for describing the behaviour of pseudoplastic fluids, followed by the Sisko model. The Power-law model is based on the fact that the relationship between shear stress and shear rate (represented in double logarithmic coordinates) can be approximated by a straight line over a limited range of values (Figure 3). The reason behind its widespread use is that in practice, a single device cannot measure the entire rheological spectrum due to a lack of sensitivity (at very low values of $\;\dot{\gamma }$) or robustness (at high values of $\;\dot{\gamma }$). In this way, the range of shear rate usually used is from 0.1 a 10^4^ s^−1^, which corresponds to the straight part of the rheogram to which the said model can be applied [16].

Thus, for this part of the flow curve, the Power-law expression is applied:

(3) $$\tau =K{\left(\dot{\gamma }\right)}^{n}\;$$

Being the apparent viscosity:

(4) $$\mu =\frac{\tau }{\dot{\gamma }}=K{\left(\dot{\gamma }\right)}^{n-1}$$

These equations involve two empirical parameters, K and n, which are known as the consistency index (measured in Pa·s*^n^*) and the flow index, respectively. If the value of n is less than 1, the fluid exhibits pseudoplastic properties. If n is equal to 1, the fluid is Newtonian, and if n is greater than 1, the fluid is dilatant. The lower the value of n, the more the fluid displays shear-thinning properties [10,12,13].

On the other hand, the Sisko model is an extension of the Power-law model that considers the viscosity value when stress rates approach infinity. As a result, it can be used in high-velocity gradient operations such as pumping liquid foods and mixing processes [17,18,19,20,21]. The mathematical expression of three parameters developed by Sisko is shown in (5):

(5) $$\tau =\mu_{\infty }\cdot \dot{\gamma }+K{\left(\dot{\gamma }\right)}^{n}$$

In this equation, the new parameter $\;\mu_{\infty }$ represents the viscosity at an infinite shear rate. Therefore, the apparent viscosity is calculated using the following expression:

(6) $$\mu =\mu_{\infty }+K{\left(\dot{\gamma }\right)}^{n-1}\;$$

Similar to the Power-law model, the values of $\;\mu_{\infty }$, K, and n are determined experimentally and are specific to each fluid.

#### 2.1.2. Shear-Thickening or Dilatant Fluids

**Figure 4:**
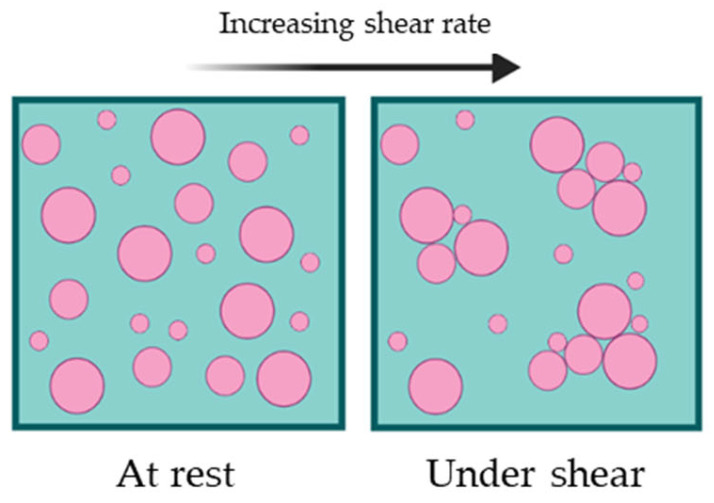
Representative diagram of the behaviour of a dilatant fluid under shear.

Dilatant fluids, in contrast to pseudoplastic fluids, demonstrate an increase in apparent viscosity as the shear rate rises. This behaviour can be explained easily: at rest, the liquid in the fluid fills the space between particles and lubricates the movement of each particle over the others at low shear levels. However, at high shear speeds, the particles pack together, blocking the passage of liquid between them, resulting in increased solid-solid friction and generating greater resistance (Figure 4) [10,12]. Dilatant fluids are commonly found in materials such as cornmeal mixed with water or fine beach sand when wet. Similar to shear-thinning fluids, dilatant fluids can be described using the Power-law or Sisko model, which have been previously mentioned.

#### 2.1.3. Viscoplastic Fluids

Viscoplastic fluids are characterized by the presence of a yield point $\tau_{0}$ that must be surpassed for the fluid to deform or flow. At rest, the material has an internal three-dimensional structure that is rigid enough to withstand external stresses that are below $\;\tau_{0}$, and it deforms elastically [10,22,23]. However, for forces that exceed $\;\tau_{0}$, the structure breaks down, and the material behaves similar to a fluid. If the relationship between shear stress and shear rate is linear, it is classified as a Bingham plastic. On the other hand, if there is no such linear relationship, it is classified as a Bingham pseudoplastic or yield-pseudoplastic [15]. In most cases, the transition from a solid-like material to a liquid-like fluid in viscoplastic materials is reversible. The extent of recovery of the structure varies for each material. Toothpaste, jam, and egg white are some examples of fluids that exhibit such behaviour.

There are also different mathematical models used to describe viscoplastic fluids, the most common being the Bingham model and the Herschel–Bulkley model, which are equivalent to the Newtonian and Power Law model, respectively, but add the yield stress [24,25]. Finally, the Casson model is a third model used for viscoplastic materials, which provides the most precise mathematical representation for studying the behaviour of biological fluids such as blood [26].

The Bingham model is the simplest equation to describe a fluid with yield stress, representing a linear relationship between shear stress and shear rate, with a displacement from the origin equal to the value of $\;\tau_{0}$. [10,14,24,27]. Hence, this model is characterized by two parameters: the yield point $\;\tau_{0}$ and the plastic viscosity $\;\mu_{B}$.

(7) $$\begin{array}{ll} \tau =\tau_{0}+\mu_{B}\left(\dot{\gamma }\right) & \;if\left|\tau \right|>\left|\tau_{0}\right|\\ \dot{\gamma }=0 & \;if\left|\tau \right|<\left|\tau_{0}\right| \end{array}$$

However, this model is not suitable for most complex fluids that do not have a linear relationship between $\dot{\gamma }$ and  τ, which can result in extremely high values of $\;\tau_{0}$ [19]. In such cases, the Herschel–Bulkley model is used, which is an adaptation of the Bingham model that includes a third parameter and provides a better fit to some experimental data.

(8) $$\begin{array}{ll} \tau =\tau_{0}+K{\left(\dot{\gamma }\right)}^{n} & \;if\left|\tau \right|>\left|\tau_{0}\right|\\ \dot{\gamma }=0 & \;if\left|\tau \right|<\left|\tau_{0}\right| \end{array}$$

Thus, the Herschel–Bulkley model is characterized by the yield point $\tau_{0}$, the flow index n and the consistency index K [24,27,28]. This model is commonly used to characterize foods such as melted chocolate, yogurt, or purées [27,29,30,31].

Lastly, the Casson model is another two-parameter model that is commonly used to describe the rheological behaviours of different types of materials. Compared to the Bingham model, the Casson model can more precisely predict the behaviour of a fluid at low shear rates [10,19,32].

(9) $$\begin{array}{ll} {\left(\tau \right)}^{1/2}={\left(\tau_{0}\right)}^{1/2}+{\left(\mu_{c}\;\dot{\gamma }\right)}^{1/2} & \;if\left|\tau \right|>\left|\tau_{0}\right|\\ \dot{\gamma }=0 & \;if\left|\tau \right|<\left|\tau_{0}\right| \end{array}$$

### 2.2. Time-Dependent Fluids

**Figure 5:**
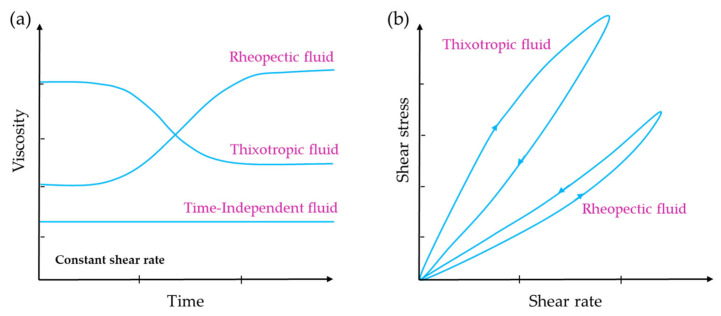
(**a**) Viscosity profiles over time at a constant shear rate and temperature. (**b**) Thixotropic loop test, showing shear stress plotted against shear rate.

Time-dependent fluids are those for which shear stress is determined by both the magnitude and the duration of the shear rate, as well as the time lapse between consecutive applications. This phenomenon is reversible, as the apparent viscosity of the fluid recovers once the shear stress is no longer applied. There are two primary categories of time-dependent fluids: thixotropic fluids and antithixotropic or rheopectic fluids. Thixotropic fluids experience a decrease in viscosity over time when subjected to a constant shear rate at a fixed temperature. However, rheopectic fluids show an increase in viscosity over time under similar conditions (Figure 5a) [33,34].

Even though the theory on time-dependent fluids is not extensively developed, a widespread agreement exists that the underlying cause of this behaviour is a reversible alteration in the fluid’s structure that takes place during the flow process [35]. In contrast to Newtonian fluids or time-independent fluids, it is not feasible to employ straightforward mathematical equations to depict the behaviour of thixotropic or rheopectic fluids. Instead, it becomes essential to conduct measurements within the desired range of conditions in order to accurately characterize their properties [10,36]. In the case of time-dependent fluids, when a flow curve develops by progressively increasing the shear rate from zero to a maximum value and subsequently decreasing it back to zero at the same rate, a hysteresis loop is observed (Figure 5b). On the other hand, for time-independent fluids, which are not influenced by time, such a loop is not observed.

#### 2.2.1. Thixotropic Fluids

As mentioned previously, thixotropic materials exhibit greater fluidity when subjected to constant shear stress over time. However, the viscosity behaviour of thixotropic materials during the process of structure decomposition and recovery differs. During shear rate acceleration, the breakdown of the structure lags behind the shear rate, resulting in transient viscosities that are higher than what would be expected at a steady state. On the descending limb of the shear rate curve, the structure continuously rebuilds as the shear rate decreases; however, the viscosities obtained are lower than those observed at steady state [33,37]. Graphically, the surface area enclosed by the hysteresis loop has been suggested as a quantitative measure of thixotropy. However, this method has notable limitations, since the height, shape, and closed area of the loop are influenced by factors such as the duration at which the shear occurs, the rate of shear increases or decreases, and the previous kinematic history of the sample. Therefore, relying solely on the loop surface area may not provide a comprehensive and accurate characterisation of the thixotropic behaviour.

Thixotropy should not be conflated with pseudoplasticity or dilatancy, which are time-independent phenomena mentioned earlier, where viscosity is directly influenced by the magnitude of the shear rate. However, it is not uncommon for a thixotropic fluid to exhibit one of these two phenomena simultaneously [36,38].

#### 2.2.2. Rheopectic Fluids

While a thixotropic fluid’s viscosity decreases over time when subjected to a constant shear rate, a rheopectic fluid’s viscosity increases when sheared. Again, hysteresis effects are observed in the flow curve (Figure 5b). However, in this case, the loop is in reverse compared to a thixotropic material. In a rheopectic fluid, shearing promotes internal structure formation; however, when the fluid is left undisturbed, it will return to its original lower viscosity [33].

### 2.3. Viscoelastic Fluids

Viscoelastic fluids possess properties that lie between those of elastic solids and purely viscous fluids. In rheology, dynamic oscillatory shear tests are a common tool used to study a broad range of complex fluids and soft matter, including but not limited to polymer solutions and melts, biological macromolecules, polyelectrolytes, surfactants, suspensions, and emulsions.

Performing dynamic oscillatory shear tests involves subjecting a material to a sinusoidal deformation and monitoring its mechanical response over time. While there are two distinct categories of oscillatory shear tests, namely large amplitude oscillatory shear (LAOS) and small amplitude oscillatory shear (SAOS), the latter has emerged as the preferred method for investigating the linear viscoelastic characteristics of these intricate fluids due to its solid theoretical foundation and the straightforward implementation of appropriate test procedures [39,40,41]. Consequently, this work will only cover these SAOS measurements. For those seeking a more comprehensive understanding of the utilization of LAOS assays, Hyun et al. published a detailed review on the subject in 2011 [40].

**Figure 6:**
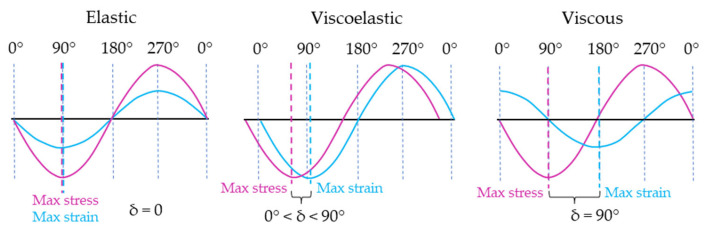
Stress and strain wave relationships for a purely elastic, viscoelastic, and purely viscous material.

The sinusoidal curves exhibit distinct behaviour depending on the mechanical characteristics of the sample, as illustrated in Figure 6. In the case of a pure elastic material, the maximum stress coincides with the maximum strain, and both stress and strain are in phase, with a phase angle (δ) of 0°. On the contrary, in a pure viscous material, the maximum stress occurs when the strain rate is at its peak, resulting in a phase shift of 90° between stress and strain. However, most samples demonstrate viscoelastic behaviour, where the phase difference between stress and strain falls between these two extremes, i.e., 0° < δ < 90° [1,42].

The ratio of the applied stress to the measured strain gives the complex modulus (G*), which is a quantitative measure of material stiffness or resistance to deformation [25].

G* = Stress*/Strain(10)

The viscoelastic behaviour of a fluid is characterised by two primary functions: the elastic or storage modulus (G′), which represents the energy accumulated by the material during each oscillation cycle, and the viscous or loss modulus (G″), which signifies the energy dissipated when the material undergoes structural changes, such as partial or complete flow of the sample. The ratio G″/G′, commonly referred to as the loss tangent or tan(δ), provides a simplified indication of whether the material exhibits solid-like or liquid-like behaviour [1,9,43].

(11) G′=G*cosδ

(12) G″=G*sinδ

It is important to avoid confusing viscoplastic materials with viscoelastic ones because of the similarity of concepts. The key rheological distinction between the two lies in the presence of a yield point ($\tau_{0}$). A viscoplastic fluid exhibits a yield stress below which it does not undergo deformation, while a viscoelastic fluid deforms under any applied stress.

## 3. Practical Assessment of the Rheological Properties of Hydrogels for 3D Printing

The investigation of a fluid’s rheology plays a crucial role in assessing its printability, specifically its suitability as a hydrogel for 3D printing via semi-solid extrusion. The resolution, shape accuracy, and reproducibility of the printing process are significantly influenced by the fluid’s behaviour. Consequently, several studies have identified parameters that have the potential to control and evaluate the printability of various materials, with a particular emphasis on rheological properties that are well suited for this type of process [9,44,45,46,47,48]. As an illustration, Yang et al. assessed the rheology of lemon juice gels with different starches by experimental studies and simulated the effect of different material properties and process parameters, such as material viscosity, inlet volume flow rate, and nozzle diameter, on the velocity and shear rate during printing. The results showed that inlet volume flow rate determined the velocity and shear velocity fields, and changes in process parameters affected the pressure field in the flow channel, with the nozzle diameter having the greatest influence [49]. Also in the field of food 3D printing, Liu et al. discovered that strong viscoelastic properties in the printed material could lead to a phenomenon known as “die swell”. This effect causes viscoelastic fluids to expand beyond the nozzle diameter when they exit the tip due to the reduction of the constraining force exerted by the tip wall. As a result, the printed samples may slightly deviate from the target geometries [50]. In a completely different domain, Blaeser et al. employed a direct fluid-dynamics model to precisely control the shear stress at the nozzle site. They conducted an extensive study to examine the impact of varying shear stress levels on cell viability and proliferation potential, aiming to achieve the printing of high-resolution and cell-viable structures. The results demonstrated that cell viability and membrane integrity were minimally affected by low rates of shear stress (<5 kPa) during the printing process, with 96% cell viability. However, higher shear stress levels (5–10 kPa and >10 kPa) resulted in a significant decrease in average cell viability, with 91% and 76% cell viability, respectively. These findings support the hypothesis that cell damage caused by the printing process is directly linked to the applied nozzle shear stress [51]. In a similar vein, He et al. performed a series of experiments and determined that pressure, feed rate and printing distance are important factors that can also influence the printing quality and cell viability [52].

**Figure 7:**
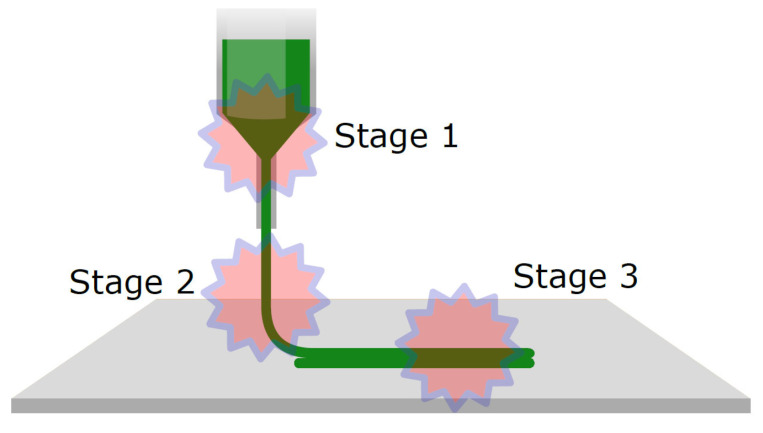
Critical stages of the extrusion process affected by the rheology of the ink.

In that way, in the context of semi-solid extrusion, several authors reported that the printability of the ink is determined by rheological properties at three critical stages, each playing a crucial role in the process (Figure 7) [9,34,44,46].

The first critical stage is located at the nozzle, just before extrusion. At this point, the rheological properties of the hydrogel are crucial. When an external force is applied to the initially at-rest fluid, its viscosity should decrease rapidly, allowing for easy extrusion through a small-diameter hole. However, once the force is removed, the viscosity must be sufficiently recovered to prevent material deposition during head movement. Therefore, it is of utmost necessity for inks to exhibit pseudoplastic behaviour, characterised by a decrease in viscosity under shear stress, and to possess a yield stress that must be surpassed for the ink to initiate flow. Highly pseudoplastic fluids require less effort to flow, enabling better control over deposition through extrusion speed (mechanical or pneumatic) and other parameters, such as nozzle diameter (smaller diameters result in higher shear rates on the fluid). The presence of a yield stress in the inks reduces the likelihood of accidental drips or unwanted deposits on the printed object. Furthermore, some researchers associate the yield point with the self-supporting capability and the formation of continuous filaments with minimal deformation [46,48,50,53,54].

At the second critical point, the hydrogel needs to exhibit a certain level of mechanical resistance once it exits the nozzle. This resistance is necessary to minimise deformation, recover a higher degree of viscosity, and ensure uniform flow. The viscoelastic nature of the fluid plays a significant role at this stage, determining whether the ink demonstrates predominantly elastic behaviour (G′ >> G″) or viscous behaviour (G″ >> G′) once it leaves the nozzle. This feature greatly influences the shape of the filament formed and consequently impacts the fidelity of the final print’s shape. Additionally, the thixotropic nature of the hydrogel is of great importance in the recovery of viscosity at this stage. Rapid thixotropic behaviour is crucial in maintaining the shape and integrity of the printed filament. It helps to control the flow of the ink, ensuring that it retains its desired form once deposited and adheres to the intended pattern during the printing process.

In a similar way, the recovery time of viscosity and the internal structure of the gel after stress also impact the third critical stage: the layer-by-layer construction. During this phase of the printing process, it is crucial for the hydrogel to rapidly develop a self-supporting capacity to withstand the weight of the subsequent layers of material being deposited on top. This self-supporting capacity ensures that the printed structure remains intact and free from deformation caused by gravity or the merging of consecutive layers [45,46]. Inks with thermosensitive behaviour are beneficial for 3D printing due to their fast-gelling speed, facilitated by the temperature difference between the extruder and the printing bed. For inks that undergo a sol-gel transition, the parameter tan(δ) is also important. Strong gels, characterised by covalent or ionic bonds, exhibit tan(δ) values below 0.1. These gels provide structural integrity to the printed object but may result in inconsistent extrusion because of gel fracturing during the nozzle passage. Weak gels have a tan(δ) greater than 0.1 and offer a more uniform extrusion but are prone to collapse after deposition unless further cross-linking is made at that point. Therefore, achieving adequate printability requires a balance between various factors [55].

To thoroughly evaluate the characteristics of a hydrogel and determine its suitability for extrusion-based 3D printing, it is essential to conduct a series of tests using a rheometer. By examining the hydrogel’s overall appearance and properties, such as its firmness, flowability, and presence of internal structures, researchers can determine the appropriate rheological testing equipment to employ. In hydrogel rheology, researchers can distinguish between a soft solid, a structured fluid, and a low-viscosity hydrogel based on the mechanical behaviour, internal structure, and flow properties. These differences can be identified through a preliminary visual assessment, which helps guide the selection of suitable testing geometries and gaps. The following sections provide an overview of the key considerations involved in this process.

### 3.1. Choosing the Right Measuring Geometry and Gap

**Figure 8:**
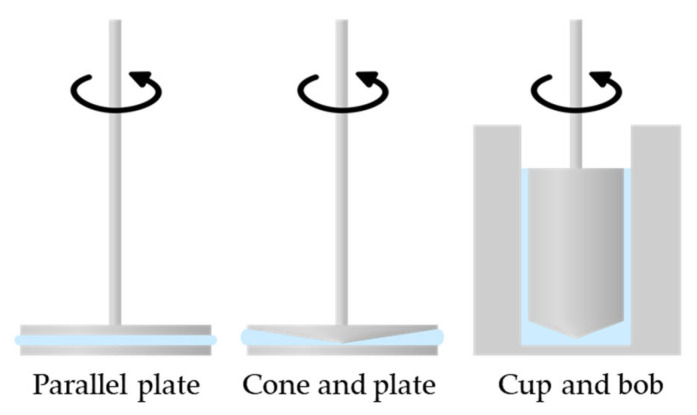
Common geometries for hydrogel rheology assessment.

The choice of the geometry of the measuring system as well as its dimensions should be carefully considered and be based on factors such as the viscosity of the hydrogel, the desired shear rate range, and the sample volume available. The standard geometries used include parallel plate, cone and plate, or cup and bob (Figure 8), but many others that come in different sizes and surface finishes can be used.

Parallel plates are simple sets of flat upper and lower plates, with sizes ranging from 4 to 60 mm in diameter, allowing for accommodation for different viscosities. The size of the gap in the rheological measurements also plays a role in determining the shear stress and the shear rate experienced by the hydrogel. For parallel plate geometries, the gap height can be adjusted to the needs of the user. Smaller gaps result in higher shear rates (at the same angular velocity), while larger gaps lead to lower shear rates. Additionally, as a general guideline, if particles are present in the hydrogel, it is recommended to select a measuring gap that is at least 10 times larger than the largest particles [25,56]. This prevents particle jamming during the measurement, which can introduce artefacts into the results.

The cone and plate combinations consist of a flat lower plate with an upper cone-shaped geometry, typically with a geometry angle of 0.5° to 4°. The smaller the cone angle, the higher the achievable shear rate. However, cone and plate geometry should be used cautiously when dealing with hydrogels that contain particles in the micrometre size range. The cone and plates have a fixed (nominal) measurement gap; for a 1° cone, the gap is 30 microns; 70 microns for 2° cones; and 150 microns for 4° [56]. This limited distance can cause particles to jam at the apex of the geometry, potentially affecting the accuracy and reliability of the measurement results [10,57]. Therefore, careful consideration should be given to the choice of measurement geometry when working with such structured hydrogels to ensure the integrity and validity of the rheological data obtained.

Finally, cup and bob geometries consist of a lower cup to hold the sample and an upper bob to measure its rheological properties. In certain cases, other accessories such as vane bobs or helical bobs can be used instead of a cylindrical bob to accommodate different fluid behaviours. Due to the large surface area, cup and bob geometries are particularly useful for low-viscosity materials and also for samples that are prone to sedimentation. Since settling occurs parallel to the geometry, the concentration of particles near the surface of the geometry remains relatively constant. This is advantageous for accurate measurements. Furthermore, the relatively large gap between the upper bob and the lower wall of the cup is beneficial when working with samples containing larger particles, as it helps prevent jamming [56,58].

In summary, there is no simple rule for selecting a geometry, as a number of factors can come into play. However, when a new sample and geometry selection are considered, the distinction between soft solids, structured fluids, and low-viscosity hydrogels may help in the selection of proper geometries and gaps.

#### 3.1.1. Low-Viscosity Hydrogels

Low-viscosity hydrogels are characterised by relatively low resistance to flow and deformation. These hydrogels have a lower overall viscosity, allowing them to flow more easily compared to soft solids or structured fluids, and also have a lower elastic modulus and a higher viscous modulus, indicating a prevalence of fluid-like behaviour. They are often used in applications where rapid and efficient flow is desired, such as injectable drug delivery systems or coatings.

When conducting rheological measurements, large-diameter cone plate or parallel plate geometries (e.g., 60 mm, 40 mm) are commonly used. In cases where the sample has a very low viscosity and is to be measured at low shear rates, it is ideal to use a measuring system with a large surface area. This allows for maximizing the torque response from the applied shear rate. However, for higher shear rate measurements, there is less need for a large surface area, since the stress and torque levels are naturally higher. In such cases, the focus shifts to avoiding turbulence, which requires the use of a geometry with a narrower gap. Narrow-angled cones or parallel plates (e.g., cone 1°/60 mm, plate 60 mm) are well-suited for this purpose, as they offer both a large surface area and a narrow gap [58]. Another option is the aforementioned cup and bob system because of the extra surface area that makes them more sensitive. However, when sample volume is limited, plate systems are generally preferred over concentric cylinders.

#### 3.1.2. Structured Fluids

Structured fluids, specifically in the context of hydrogels, refer to substances that exhibit fluid-like behaviour while still maintaining a certain level of internal structure. Structured fluids possess the ability to flow and deform under applied stress, similar to a fluid, but they also retain a degree of structural organization. They have a lower elastic modulus compared to soft solids and a higher viscous modulus, indicating a greater influence of fluid-like behaviour. Structured fluids are commonly used in applications where controlled flow, shear thinning, or shape recovery are desired, such as drug delivery systems or printable inks for 3D bioprinting.

These fluids can also contain particles, droplets, or air bubbles, which further contribute to their complex behaviour. When it comes to rheological testing, common accessories used for structured fluids include parallel plates of various sizes (40 mm, 25 mm, or 20 mm), or a cup combined with a vane rotor or a helical rotor to avoid slippage.

#### 3.1.3. Soft Solids

A soft solid refers to a hydrogel that exhibits solid-like characteristics under certain conditions. It possesses a defined structure that enables it to resist deformation and maintain its shape, similar to a solid material. Soft solids have a significant elastic component in their rheological response, meaning they can store and recover energy upon deformation. They typically exhibit a high elastic modulus (G′) compared to their viscous modulus (G″), indicating a predominance of solid-like behaviour. Soft solids are often used in applications where mechanical strength and structural integrity are crucial, such as load-bearing tissues or scaffold materials for tissue engineering. The use of a parallel plate geometry in its smaller size (<25 mm) is recommended for testing soft solids [25,56]. Specifically, it is beneficial to employ a roughened surface on the parallel plate, such as crosshatched or sandblasted plates, to prevent slippage and ensure accurate measurements. Again, cup and vane/helical rotor systems can be used for the same reason.

### 3.2. Selecting and Setting the Correct Test Methods

Hydrogels are versatile materials that can also undergo transitions between these categories because of their physical properties and chemical compositions. For instance, when the gelling of a hydrogel is temperature dependent, it can exist in a low-viscosity liquid phase and be transformed into a structured fluid (or even a soft solid) with a temperature change. Similarly, crosslinking processes (such as chemical, light, pH-dependent, or enzymatic) can convert a low viscosity hydrogel or a structured fluid into a final soft solid with mechanical properties that are better suited for its intended application. These transitions highlight the dynamic nature of hydrogels and their ability to adapt their physical properties based on external stimuli or internal changes, making them highly versatile materials in various fields, including biomedicine, drug delivery, or tissue engineering. However, those transitions also reflect a need to define the appropriate testing protocols as an essential tool to accurately characterize the hydrogel for its intended use and printing conditions.

Different tests, including flow curves, oscillatory tests, and creep/recovery tests, can provide valuable insights into the viscoelastic properties, flow behaviour, or time-dependent response of the hydrogel, among others. The selection of tests should be based on the specific rheological characteristics that play a crucial role in achieving a successful 3D printing and final product, such as the ink’s shear thinning behaviour, elastic modulus, and recovery time. The range and resolution of these parameters should be adjusted to match the expected rheological properties of the hydrogel during printing and during its final use.

#### 3.2.1. Fluid Behaviour and Yield Stress Determination

The fundamental test in rheology involves measuring viscosity and shear stress by varying the shear rate within a specific range while maintaining a constant temperature, typically between 10^−2^ and 10^3^ s^−1^ for hydrogels. The measured shear rate can be compared with the typical ranges of shear rates for common industrial processes. For example, low shear rates (10^−2^–10^−1^ s^−1^) provide information on fluid behaviour in processes such as storage, levelling, or sedimentation, which are useful for assessing the stability of a hydrogel at rest. The range between 10^−1^ and 10^3^ s^−1^ represents physically active processes such as mixing, stirring, pumping, or extruding, which helps determine the suitability of the hydrogel for extrusion-based 3D printing. Shear rates greater than 10^4^ s^−1^ are associated with spraying, brushing, or high-speed coatings, which are not commonly applicable to hydrogels suitable for this 3D printing technology [59].

This test generates data that can be used to plot a flow curve, providing insight into the material’s behaviour (whether it exhibits pseudoplastic, dilatant, or viscoplastic properties). By analysing the data obtained, the most appropriate mathematical model can be selected to extract and interpret the model parameters. For instance, Li et al. employed the Power-law model and experimental data to assess the shear-thinning characteristics of various compositions and determine the shear rate experienced by the hydrogel during the printing process [60]. Other studies have utilized curve fitting with mathematical models to evaluate specific parameters such as the yield stress [61,62]. While fitting a flow curve to a mathematical model is one of the simplest methods to evaluate $\;\tau_{0}$, as explained later, it is not the only approach.

#### 3.2.2. Amplitude Sweeps and Determination of LVR, *γ_c_* and $\tau_{0}$

To measure viscoelasticity, two commonly performed small amplitude oscillatory tests (SAOS) are used to evaluate the two main parameters: the storage modulus (G′) and the loss modulus (G″). These tests are amplitude sweeps and frequency sweeps.

Amplitude sweeps are obtained by subjecting the sample to a range of deformations (from 10^−1^–10^3^%) at a fixed frequency (typically between 0.1 and 1 Hz for hydrogels). It is very rare to require values above or below this range for measuring hydrogels suitable for 3D extrusion printing. However, the values can be adjusted on the basis of the initial characteristics of the sample (viscosity, presence of particles, level of crosslinking, etc.) if necessary. The measurement results are typically presented in a diagram where strain (or shear stress) is plotted on the x-axis, and storage modulus (G′) and loss modulus (G″) are plotted on the y-axis, with both axes displayed on a logarithmic scale. This representation helps to estimate the linear viscoelastic region (LVR).

**Figure 9:**
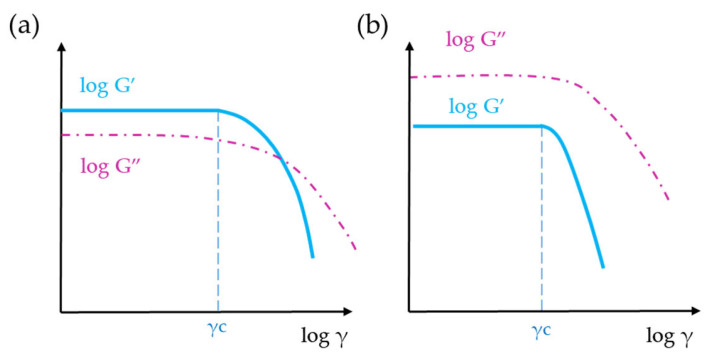
Diagram of two amplitude sweeps showing the LVR: (**a**) gel-like sample (G′ > G″); (**b**) fluid-like sample (G″ > G′).

The LVR indicates the range in which an oscillatory rheology test can be performed without destroying the structure of the sample. The limit of the LVR, also known critical strain (*γ_c_*), is the amplitude value at which the storage modulus values cease to be approximately constant. *γ_c_* is typically determined as the first point at which G′ reaches a value equal to or lower than 95–90% of its initial value [63,64]. The extent of LVR is inversely proportional to the solid character of the sample and is sensitive to frequency and temperature [43,57,65,66]. Furthermore, it is common to evaluate the values of G′ and G″ in the linear viscoelastic region because this analysis provides insight into the viscoelastic nature of the sample. When G′ > G″, the sample exhibits a gel-like or solid structure, indicating a viscoelastic solid material (Figure 9a). Conversely, when G″ > G′, the sample demonstrates a fluid-like structure, indicating a viscoelastic liquid (Figure 9b). It is important to note that these classifications are specific to the applied measuring conditions, particularly to the preset (angular) frequency.

**Figure 10:**
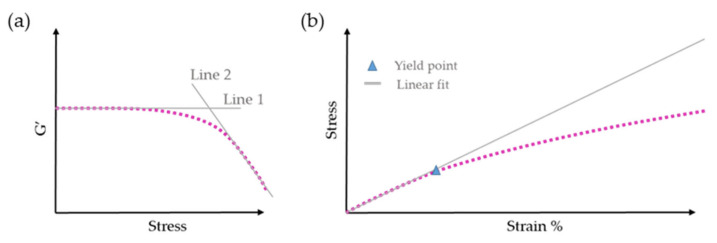
Methods for yield stress detection using amplitude sweeps: (**a**) intersection of the tangents of the storage modulus (G′); (**b**) point of linearity deviation in the stress—strain% curve. The pink dotted line represents the experimental results.

Stress sweeps can also be useful in determining the yield stress when it is not possible to do so through flow curves, such as in the case of materials with high viscosity at room temperature. In these types of fluid, an increase in shear stress results in sample slippage between plates, and the measured yield stress is lower than the actual yield stress of the sample [65,67]. In an amplitude sweep, $\tau_{0}\;$ can be estimated using two different methods. The first method involves determining the intersection of the tangents of the storage modulus at high and low oscillatory stresses (Figure 10a). The first line is fitted to the values within the LVR, and the second line is derived from the storage modulus measured for stresses exceeding the LVR [68,69]. However, there is some ambiguity in choosing the number of points required to obtain the second line, which can reduce the reliability of the method.

In the second method, a plot of stress (Pa) versus oscillatory strain (%) is used (Figure 10b). The stress-strain curve is linear at low strain because the fluid response is predominantly elastic. The point at which the curve deviates from linearity is determined as the yield stress [68,69]. It is important to note that the value of $\tau_{0}$ obtained differs depending on the method used, sometimes called the apparent yield stress [67].

#### 3.2.3. Frequency Sweeps and Determination of tan(δ)

**Figure 11:**
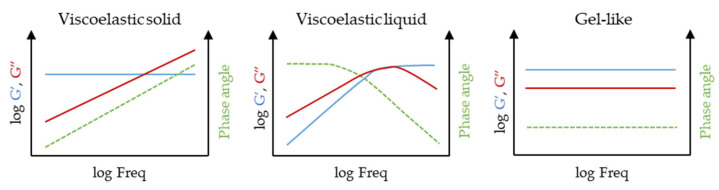
Frequency sweeps at constant temperature that can be found in hydrogels. Blue lines represent the storage modulus (G’), red lines represent the loss modulus (G”) and green lines represent the phase angle.

As mentioned above, the second SAOS test most commonly performed is the frequency sweep. In this case, the strain amplitude is kept constant and within the LVR, while the oscillation frequency is increased. This test helps to better understand the internal structure of the material and its time-dependent behaviour in the non-destructive deformation range. For example, high frequencies represent short-term behaviours, such as those occurring in a mixing or extrusion process, while low frequencies represent long-term behaviours, such as settling or resting [46,68]. Figure 11 illustrates the frequency sweep measurement of different materials, including viscoelastic solids, viscoelastic liquids, and gel-like materials. In the case of viscoelastic solids, the storage modulus G′ remains constant and dominates at low frequencies. However, as the frequency increases, the loss modulus G″ becomes more prominent. These materials exhibit a firm consistency when at rest but can be easily deformed under sufficient force. Viscoelastic liquid materials, on the other hand, demonstrate a different behaviour. At low frequencies, the loss modulus G″ surpasses the storage modulus G′, indicating liquid-like behaviour. As the frequency increases, the storage modulus G′ becomes higher than G″, suggesting a solid-like behaviour. For gel-like materials, both G′ and G″ remain parallel across the entire frequency range. This indicates that the material maintains consistent behaviour regardless of the frequency applied. Gels often exhibit a solid-like structure while retaining some fluid-like properties.

Frequency sweeps can be used to evaluate the value of tan(δ) for each fluid and its variability with respect to angular frequency. Practically, the value of tan(δ) serves as a significant criterion for analysing hydrogel formation, hardening, and curing processes. In the fluid or liquid state (“sol state”), tan(δ) is greater than 1 (as G″> G′), indicating a more liquid-like behaviour. On the contrary, in the gel-like or solid state, tan(δ) is less than 1 (as G′> G″), reflecting solid-like characteristics. At the point of maintaining the sol-gel transition, tan(δ) has a value equal to 1 [1,70]. In this context, tan(δ) has been employed as a parameter to assess the balance between viscous flow and elasticity, which is crucial for extrusion-based 3D printing. For instance, Petta et al. established a correlation between tan(δ) values and the extrudability of tyramine hyaluronan derivative hydrogels, indicating that formulations with tan(δ) values ranging from 0.5 to 0.6 exhibited the optimal printability for their printing conditions [71]. Similarly, Cheng et al. highlighted that for the specific 3D printing platform used by their team, the printing material should ideally have a tan(δ) ranging from 0.2 to 0.7 [72].

#### 3.2.4. Thixotropy and Viscosity Recovery

As stated above, thixotropy is a time-dependent property that characterizes the reversibility of changes occurring in the internal structure of a gel. In thixotropic fluids, the apparent viscosity decreases when a constant shear rate is applied over time and gradually recovers when the force on the fluid decreases or ceases. This phenomenon is measured through recovery tests at different shear rates and a constant temperature, known as the Stepped Flow Method (SFM) or the Stepped Dynamic Method (SDM), depending on whether the test is performed using linear or oscillatory rheology [57,73].

**Figure 12:**
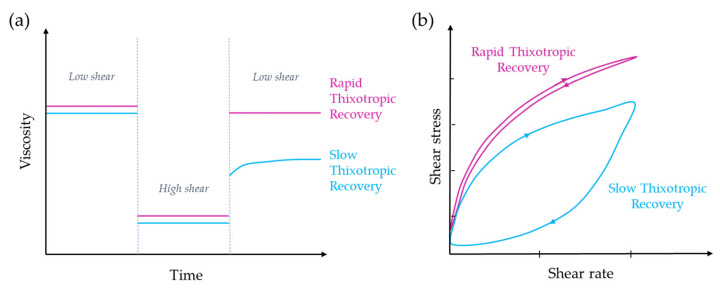
Thixotropy evaluation diagrams: (**a**) viscosity recovery evaluation by the SFM/SDM method, (**b**) thixotropy loop.

These tests involve three distinct stages that examine the behaviour of the fluid over time by applying different shear rates or deformations (Figure 12a). In the SFM test, the initial stage entails the application of a low shear rate (around 0.4 s^−1^) for a relatively extended period (60 to 180 s). Subsequently, a high shear rate (around 100 s^−1^) is implemented, resulting in disruption of the internal gel structure and a significant reduction in viscosity for a brief duration (20–40 s). Finally, the sample undergoes a recovery stage with a low shear rate lasting an additional 120–180 s [46,48]. Similarly, in the SDM test, the stages remain the same, but the amplitude of the oscillation varies. The amplitude is set at a low level during the first and third stages (approximately 0.1% strain or an appropriate value within the linear viscoelastic region, LVR). In contrast, during the second stage, the amplitude is adjusted to a sufficiently high level, exceeding the LVR, to induce the destruction of the colloidal gel network [57,61]. By manipulating the applied stresses on the material in this manner, it becomes possible to evaluate the extent and nature of the internal structure destruction, as well as determine the percentage of regeneration at various time intervals. From a practical standpoint, the percentages of viscosity recovery can be calculated using numerical methods. Viscosity recovery refers to the percentage of viscosity achieved during the initial 30 s and/or the final 60 s of the third step (after undergoing high deformation), based on the average viscosity obtained during the last 30 s of the initial step.

Additionally, thixotropic fluids can be categorized based on their viscosity recovery rate after the shear stress is removed. A rapid thixotropic fluid quickly regains its viscosity, making it ideal for applications that require precise control over material flow and shape, such as 3D printing. In contrast, a slow thixotropic fluid takes more time to restore its original viscosity, resulting in prolonged spreading or deformation. In this way, Paxton et al. conducted a comparative analysis of viscosity recovery in hydrogels, specifically Poloxamer 407 and alginate, emphasizing the significance of rapid viscosity recovery for 3D printing. The printable concentrations of 25 wt% and 30 wt% Poloxamer 407 exhibited fast recovery, ensuring shape fidelity. Conversely, the unprintable 15 wt% Poloxamer 407 took longer to stabilize, resulting in reduced shape retention. All Poloxamer 407 samples fully regained their initial viscosity within 200 s, indicating no permanent changes in polymer structure or properties due to the high shear conditions of bioprinting. Similarly, the 8% *w*/*v* alginate sample demonstrated satisfactory recovery over the entire 200 s period, albeit with a slight delay in viscosity recovery observed by the slope in the curve after transitioning from high to low shear rate. In contrast, the pre-crosslinked alginate sample exhibited more rapid recovery, further supporting its suitability as a printable ink compared to the uncrosslinked sample [48]. Nevertheless, other studies demonstrate cases where fluids with slower recovery times can still be successfully printed by incorporating temperature-induced gelation or utilizing in situ rapid crosslinking reactions during the printing process [45,74]. Thus, thixotropic analysis allows researchers to determine if their material may require additional crosslinking processes to increase rapidly viscosity after extrusion. In that sense, thixotropy loops can also provide valuable information (Figure 12b). If the sample exhibits rapid thixotropic recovery, the downsweep curve will closely overlay the upsweep curve, indicating a minimal time-dependent delay in recovery. However, if the thixotropic recovery is slow or the sample has potentially been damaged, the downsweep curve will deviate from the expected path. The extent of deviation indicates the duration of recovery or the severity of potential damage, with larger deviations indicating longer recovery times or more significant damage [73]. This delayed viscosity recovery presents challenges to maintaining precise control over flow and shape.

#### 3.2.5. Gelation Kinetics and Gel Point Determination

Hydrogels can be formed through either physical polymer chain association or different cross-linking reactions. The gelation mechanism employed significantly impacts the mechanical and thermal properties of the hydrogel. To ensure consistent properties of the hydrogel for a particular application, it is crucial to comprehend the chemistry of gelation and quantitatively analyse the physical properties of the gel. Rheological techniques can be employed to determine the gel point, enabling the use of a rheometer to measure the kinetics of gelation.

**Figure 13:**
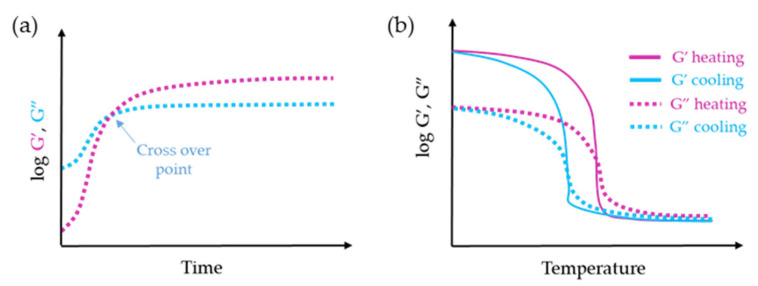
Gelation kinetics diagrams: (**a**) isothermal hydrogel cross-linking reaction, (**b**) temperature ramp.

In a curing system, the gel point can be utilized to monitor the progress of the curing reaction and determine the kinetics of gelation. The commonly employed method for determining the gelation point involves conducting isothermal dynamic time sweep experiments at a fixed temperature. Oscillatory shear is continuously applied at fixed strain deformation in the LVR to monitor gelation at a sampling interval of 20–30 s. Throughout the measurement, the changes in modulus (G′, G″, and G*) and complex viscosity are continuously monitored over time, recording the sol-gel transition time, which is the point at which G′ crosses over G″ [75,76,77]. This transition time can vary depending on factors such as the concentration of the crosslinking agent and the reaction temperature. Once gel formation occurred, the G′ and G″ curves reached plateaus, indicating the completion of the crosslinking reaction (Figure 13a). Additionally, in their work, Ravanbakhsh et al. mentioned an alternative method for determining the gelation point that involves identifying the point at which the ratio between G′ and G″ becomes independent of frequency [78].

For thermal reversible hydrogels, the gelation and gel-melting process can be assessed using dynamic temperature ramp tests. These tests involve gradually decreasing the temperature (ramp down) or increasing it (ramp up) at rates typically around 1 °C/min and 2 °C/min, respectively. During the cooling process, the hydrogel solution undergoes a transition from a liquid to a solid state, indicated by the crossover of the elastic modulus (G′) and viscous modulus (G”) at small range of temperatures (Figure 13b). It is worth noting that the mechanical strength of the sample, represented by the elastic modulus (G′), may increase by more than five orders of magnitude during the gelation process. On the other hand, after heating, the hydrogel starts to melt at values where G” > G′. Thus, this temperature-raising procedure is an effective means to monitor the gelation process of a thermally reversible hydrogel [57,79].

To conduct these tests and analyse the viscoelastic properties of hydrogels, a rotational rheometer equipped with a temperature control system is required. Additionally, to prevent slippage during measurements, the use of a roughened plate, such as a sandblasted or crosshatched plate, is recommended. The measurements should be performed within the linear viscoelastic region of the material, typically at a constant frequency of 1 Hz [57,79]. 

#### 3.2.6. Gel Strength

The mechanical properties in terms of stiffness of 3D structures can be accurately examined using oscillatory rheology. The modulus of elasticity (G′) and, therefore, the stiffness of a sample can be determined through two types of oscillatory tests: amplitude sweeps, where the hydrogel is subjected to a range of deformations (strain %) at a fixed frequency as mentioned earlier, or dynamic frequency sweeps, where the sample is subjected to a specific frequency range at a fixed strain percentage. Both tests are carried out at a constant temperature [57,80].

This test is particularly relevant in the case of hydrogels used in bioprinting, as the biomechanical properties of tissues vary significantly between organs and tissues and are inherently related to their function. Mechanically static tissues, such as the brain, and adaptable tissues, such as the lung, exhibit low rigidity. On the other hand, tissues exposed to high mechanical loads, such as bone or skeletal muscle, have elastic moduli that are several orders of magnitude higher [81]. In this regard, it has been demonstrated that the biochemical and biomechanical properties of inks influence cellular and tissue compatibility [74,82,83]. For example, studies conducted with chondrocytes have revealed that the rigidity of the hydrogel that encapsulates them is important for the distribution, organization, and secretion of type II collagen, which is essential for repairing articular lesions [84]. Other reports have shown that cells proliferate faster when grown on the surface of high-strength hydrogels. However, when cells are cultured in a 3D microenvironment, their survival and proliferation decrease as the matrix strength increases [85]. Therefore, it is advisable to perform this test on inks involved in bioprinting processes whose firmness can be modulated through cross-linking reactions and come into contact with cellular loads: (i) directly, by containing cells embedded within them or serving as a support or scaffold for cell seeding, or (ii) indirectly, by being designed for implantation in the body.

One of the most common protocols for the practical assessment of the stiffness of formulated and/or printed systems is described by Cox and Madsen [80]. In this protocol, samples are subjected to stress sweeps with a small range of deformation (approximately 0.2% to 2%), allowing the extraction of the storage modulus (G′) at 1%. This enables the comparison of multiple measurements within the same ink, multiple inks, multiple structures, or any combination of interest. Such tests can also be employed to evaluate the crosslinking of hydrogels. For example, they can be conducted at different concentrations of cross-linking agents or exposure times, regardless of the type of cross-linking that occurs [86].

## 4. Conclusions

In conclusion, the field of rheology plays a vital role in the extrusion-based 3D printing of hydrogels, where the material’s flow and deformation properties significantly impact its behaviour during the printing process. The rheological properties, including viscosity, shear thinning behaviour, and viscoelasticity, are crucial factors for determining the extrudability, shape retention, and overall quality of the printed structures. Understanding these properties is essential to optimising the printing process and achieving the desired accuracy and quality of the final product. A thorough knowledge of the rheological concepts, terminology, and tests applicable to 3D printing facilitates the evaluation of the printability of different hydrogels and has the potential to drive progress in the fields of tissue engineering, drug delivery, and regenerative medicine. By bridging the gap between rheology and hydrogel 3D printing, researchers and scientists from diverse disciplines can unlock new opportunities for innovation and application within this interdisciplinary field.

## Data Availability

Data sharing is not applicable to this article.

## References

[1] Mezger T.G. (2006). The Rheology Handbook: For Users of Rotational and Oscillatory Rheometers.

[2] Murata H. (2012). Rheology—Theory and Application to Biomaterials. Polymerization.

[3] Tajik S., Garcia C.N., Gillooley S., Tayebi L. (2023). 3D Printing of Hybrid-Hydrogel Materials for Tissue Engineering: A Critical Review. Regen. Eng. Transl. Med..

[4] Hu F., Mikolajczyk T., Pimenov D.Y., Gupta M.K. (2021). Extrusion-Based 3D Printing of Ceramic Pastes: Mathematical Modeling and In Situ Shaping Retention Approach. Materials.

[5] Kaliaraj G., Shanmugam D., Dasan A., Mosas K. (2023). Hydrogels—A Promising Materials for 3D Printing Technology. Gels.

[6] Amorim S., Soares da Costa D., Pashkuleva I., Reis C.A., Reis R.L., Pires R.A. (2021). 3D Hydrogel Mimics of the Tumor Microenvironment: The Interplay among Hyaluronic Acid, Stem Cells and Cancer Cells. Biomater. Sci..

[7] Kadajji V.G., Betageri G.V. (2011). Water Soluble Polymers for Pharmaceutical Applications. Polymers.

[8] Díaz A., Herrada-Manchón H., Nunes J., Lopez A., Díaz N., Grande H., Loinaz I., Fernández M.A., Dupin D. (2022). 3D Printable Dynamic Hydrogel: As Simple as It Gets!. Macromol. Rapid Commun..

[9] Schwab A., Levato R., D’Este M., Piluso S., Eglin D., Malda J. (2020). Printability and Shape Fidelity of Bioinks in 3D Bioprinting. Chem. Rev..

[10] Chhabra R.P., Richardson J.F. (1999). Non-Newtonian Flow in the Process Industries—Fundamentals and Engineering Applications.

[11] Walters K., Hutton J.F., Barnes H.A., Walters K. (1993). An Introduction to Rheology.

[12] Ramírez-Navas J.S. (2006). Introducción a La Reología de Alimentos. Rev. ReCiTeIA.

[13] Masoliver i Marcos G., Pérez-Sánchez M., López-Jiménez P.A. (2017). Modelo Experimental Para Estimar La Viscosidad de Fluidos No Newtonianos: Ajuste a Expresiones Matemáticas Convencionales. Model. Sci. Educ. Learn..

[14] Zengeni B.T. (2016). Bingham Yield Stress and Bingham Plastic Viscosity of Homogeneous Non-Newtonian Slurries. Ph.D. Thesis.

[15] Chhabra R.P., Richardson J.F. (2008). Non-Newtonian Fluid Behaviour. Non-Newtonian Flow and Applied Rheology: Engineering Applications.

[16] Lakkanna M., Mohan Kumar G.C., Kadoli R. (2016). Computational Design of Mould Sprue for Injection Moulding Thermoplastics. J. Comput. Des. Eng..

[17] Nindo C.I., Tang J., Powers J.R., Takhar P.S. (2007). Rheological Properties of Blueberry Puree for Processing Applications. LWT-Food Sci. Technol..

[18] Quintáns Riveiro L.C. (2008). Reología de Productos Alimentarios. Ph.D. Thesis.

[19] Li M.-C., Wu Q., Song K., De Hoop C.F., Lee S., Qing Y., Wu Y. (2016). Cellulose Nanocrystals and Polyanionic Cellulose as Additives in Bentonite Water-Based Drilling Fluids: Rheological Modeling and Filtration Mechanisms. Ind. Eng. Chem. Res..

[20] TA Instruments Rheology Aplications Note: Rheology Software Models (Flow). http://www.tainstruments.com/pdf/literature/RN9.pdf.

[21] Sisko A.W. (1958). The Flow of Lubricating Greases. Ind. Eng. Chem..

[22] Balmforth N.J., Frigaard I.A., Ovarlez G. (2014). Yielding to Stress: Recent Developments in Viscoplastic Fluid Mechanics. Annu. Rev. Fluid Mech..

[23] El-Borhamy M. (2018). Numerical Study of the Stationary Generalized Viscoplastic Fluid Flows. Alexandria Eng. J..

[24] Bird R.B., Dai G.C., Yarusso B.J. (1983). The Rheology and Flow of Viscoplastic Materials. Rev. Chem. Eng..

[25] Chen T. Rheology Basic Theory & Applications Training Section #1. https://www.tainstruments.com/wp-content/uploads/2020-Rheology-Online-Training-1.pdf.

[26] Oke A.S., Mutuku W.N., Kimathi M., Animasaun I.L. (2020). Insight into the Dynamics of Non-Newtonian Casson Fluid over a Rotating Non-Uniform Surface Subject to Coriolis Force. Nonlinear Eng..

[27] Adewale F.J., Lucky A.P., Oluwabunmi A.P., Boluwaji E.F. (2017). Selecting the Most Appropriate Model for Rheological Characterization of Synthetic Based Drilling Mud. Int. J. Appl. Eng. Res..

[28] Talens Oliag P. (2016). Caracterización Del Comportamiento Reológico de Un Alimento Fluido Plástico. RiuNet Repos. UPV..

[29] Fernández C., Alvarez M.D., Canet W. (2004). Rheological Behaviour of Fresh and Frozen Potato Puree in Steady and Dynamic Shear at Different Temperatures. Eur. Food Res. Technol..

[30] Fernández C., Canet W., Alvarez M.D. (2009). Quality of Mashed Potatoes: Effect of Adding Blends of Kappa-Carrageenan and Xanthan Gum. Eur. Food Res. Technol..

[31] Sokmen A., Gunes G. (2006). Influence of Some Bulk Sweeteners on Rheological Properties of Chocolate. LWT-Food Sci. Technol..

[32] HadjSadok A., Moulai-Mostefa N., Rebiha M. (2010). Rheological Properties and Phase Separation of Xanthan-Sodium Caseinate Mixtures Analyzed by a Response Surface Method. Int. J. Food Prop..

[33] TA Instruments Understanding Rheology of Structured Fluids. http://www.tainstruments.com/pdf/literature/AAN016_V1_U_StructFluids.pdf.

[34] Cooke M.E., Rosenzweig D.H. (2021). The Rheology of Direct and Suspended Extrusion Bioprinting. APL Bioeng..

[35] Sochi T. (2010). Non-Newtonian Flow in Porous Media. Polymer.

[36] Mewis J., Wagner N.J. (2011). Colloidal Suspension Rheology.

[37] Armelin E., Martí M., Rudé E., Labanda J., Llorens J., Alemán C. (2006). A Simple Model to Describe the Thixotropic Behavior of Paints. Prog. Org. Coat..

[38] Sha J., Zhang F., Zhang H. (2016). Thixotropic Flow Behaviour in Chemical Pulp Fibre Suspensions. BioResources.

[39] Malvern Instruments A Basic Introduction to Rheology. https://cdn.technologynetworks.com/TN/Resources/PDF/WP160620BasicIntroRheology.pdf.

[40] Hyun K., Wilhelm M., Klein C.O., Cho K.S., Nam J.G., Ahn K.H., Lee S.J., Ewoldt R.H., McKinley G.H. (2011). A Review of Nonlinear Oscillatory Shear Tests: Analysis and Application of Large Amplitude Oscillatory Shear (LAOS). Prog. Polym. Sci..

[41] Tschoegl N.W. (1989). The Phenomenological Theory of Linear Viscoelastic Behavior.

[42] Ramli H., Zainal N.F.A., Hess M., Chan C.H. (2022). Basic Principle and Good Practices of Rheology for Polymers for Teachers and Beginners. Chem. Teach. Int..

[43] Trujillo L.A., Santos J., Calero N., Alfaro M.C., Muñoz J. (2013). Caracterización Reológica de Una Suspoemulsión Comercial Para Uso Agroquimico. Afinidad.

[44] Kyle S., Jessop Z.M., Al-Sabah A., Whitaker I.S. (2017). ‘Printability’ of Candidate Biomaterials for Extrusion Based 3D Printing: State-of-the-Art. Adv. Healthc. Mater..

[45] Diañez I., Gallegos C., Brito-de la Fuente E., Martínez I., Valencia C., Sánchez M.C., Diaz M.J., Franco J.M. (2019). 3D Printing in Situ Gelification of κ-Carrageenan Solutions: Effect of Printing Variables on the Rheological Response. Food Hydrocoll..

[46] Liu Z., Bhandari B., Prakash S., Mantihal S., Zhang M. (2019). Linking Rheology and Printability of a Multicomponent Gel System of Carrageenan-Xanthan-Starch in Extrusion Based Additive Manufacturing. Food Hydrocoll..

[47] Moore C.A., Shah N.N., Smith C.P., Rameshwar P. (2018). 3D Bioprinting and Stem Cells. Methods in Molecular Biology.

[48] Paxton N., Smolan W., Böck T., Melchels F., Groll J., Jungst T. (2017). Proposal to Assess Printability of Bioinks for Extrusion-Based Bioprinting and Evaluation of Rheological Properties Governing Bioprintability. Biofabrication.

[49] Yang F., Guo C., Zhang M., Bhandari B., Liu Y. (2019). Improving 3D Printing Process of Lemon Juice Gel Based on Fluid Flow Numerical Simulation. LWT.

[50] Liu Z., Bhandari B., Prakash S., Zhang M. (2018). Creation of Internal Structure of Mashed Potato Construct by 3D Printing and Its Textural Properties. Food Res. Int..

[51] Blaeser A., Duarte Campos D.F., Puster U., Richtering W., Stevens M.M., Fischer H. (2016). Controlling Shear Stress in 3D Bioprinting Is a Key Factor to Balance Printing Resolution and Stem Cell Integrity. Adv. Healthc. Mater..

[52] He Y., Yang F., Zhao H., Gao Q., Xia B., Fu J. (2016). Research on the Printability of Hydrogels in 3D Bioprinting. Sci. Rep..

[53] Smith P.T., Basu A., Saha A., Nelson A. (2018). Chemical Modification and Printability of Shear-Thinning Hydrogel Inks for Direct-Write 3D Printing. Polymer.

[54] Sun A., Gunasekaran S. (2009). Yield Stress in Foods: Measurements and Applications. Int. J. Food Prop..

[55] Saha D., Bhattacharya S. (2010). Hydrocolloids as Thickening and Gelling Agents in Food: A Critical Review. J. Food Sci. Technol..

[56] NETZSCH-Gerätebau GmbH Rheology—How to Select the Appropriate Measuring Geometry. https://analyzing-testing.netzsch.com/en/training-know-how/tips-tricks/rheology/rheology-how-to-select-the-appropriate-measuring-geometry.

[57] Chen T. Rheology: Basic Theory and Applications Training Section #2. https://www.tainstruments.com/wp-content/uploads/2020-Rheology-Online-Training-2.pdf.

[58] Rolfe P. Viscosity Flow Curve. Part 2. How to Best Measure a Viscosity Flow Curve?. https://www.materials-talks.com/blog/2017/06/08/viscosity-flow-curve-part-2/.

[59] Carnicer V., Alcázar C., Orts M.J., Sánchez E., Moreno R. (2021). Microfluidic Rheology: A New Approach to Measure Viscosity of Ceramic Suspensions at Extremely High Shear Rates. Open Ceram..

[60] Li H., Liu S., Lin L. (2016). Rheological Study on 3D Printability of Alginate Hydrogel and Effect of Graphene Oxide. Int. J. Bioprint..

[61] Herrada-Manchón H., Rodríguez-González D., Fernández M.A., Kucko N.W., Barrère-de Groot F., Aguilar E. (2022). Effect on Rheological Properties and 3D Printability of Biphasic Calcium Phosphate Microporous Particles in Hydrocolloid-Based Hydrogels. Gels.

[62] Haring A.P., Thompson E.G., Tong Y., Laheri S., Cesewski E., Sontheimer H., Johnson B.N. (2019). Process- and Bio-Inspired Hydrogels for 3D Bioprinting of Soft Free-Standing Neural and Glial Tissues. Biofabrication.

[63] Kreimendahl F., Köpf M., Thiebes A.L., Duarte Campos D.F., Blaeser A., Schmitz-Rode T., Apel C., Jockenhoevel S., Fischer H. (2017). Three-Dimensional Printing and Angiogenesis: Tailored Agarose-Type I Collagen Blends Comprise Three-Dimensional Printability and Angiogenesis Potential for Tissue-Engineered Substitutes. Tissue Eng. Part C Methods.

[64] TA Instruments Determining the Linear Viscoelastic Region in Oscillatory Measurements. https://www.tainstruments.com/pdf/literature/RH107.pdf.

[65] Anton Paar GmbH Amplitude Sweeps. https://wiki.anton-paar.com/en/amplitude-sweeps/.

[66] Talens Oliag P. (2018). Cómo Caracterizar El Comportamiento Viscoelástico de Un Alimento. https://riunet.upv.es/bitstream/handle/10251/103393/Talens%20-%20C%C3%B3mo%20caracterizar%20el%20comportamiento%20viscoel%C3%A1stico%20de%20un%20alimento.pdf?sequence=1.

[67] TA Instruments Rheological Techniques for Yield Stress Analysis. https://www.tainstruments.com/pdf/literature/RH025.pdf.

[68] Huang C.Y. (2018). Extrusion-Based 3D Printing and Characterization of Edible Materials. Master’s Thesis.

[69] Cyriac F., Lugt P.M., Bosman R. (2015). On a New Method to Determine the Yield Stress in Lubricating Grease. Tribol. Trans..

[70] Duty C., Ajinjeru C., Kishore V., Compton B., Hmeidat N., Chen X., Liu P., Hassen A.A., Lindahl J., Kunc V. (2018). What Makes a Material Printable? A Viscoelastic Model for Extrusion-Based 3D Printing of Polymers. J. Manuf. Process..

[71] Petta D., Grijpma D.W., Alini M., Eglin D., D’Este M. (2018). Three-Dimensional Printing of a Tyramine Hyaluronan Derivative with Double Gelation Mechanism for Independent Tuning of Shear Thinning and Postprinting Curing. ACS Biomater. Sci. Eng..

[72] Cheng Y., Qin H., Acevedo N.C., Jiang X., Shi X. (2020). 3D Printing of Extended-Release Tablets of Theophylline Using Hydroxypropyl Methylcellulose (HPMC) Hydrogels. Int. J. Pharm..

[73] TA Instruments Introduction to Thixotropy Analysis Using a Rotational Rheometer. https://www.tainstruments.com/pdf/literature/RH106.pdf.

[74] Herrada-Manchón H., Celada L., Rodríguez-González D., Alejandro Fernández M., Aguilar E., Chiara M.-D. (2021). Three-Dimensional Bioprinted Cancer Models: A Powerful Platform for Investigating Tunneling Nanotube-like Cell Structures in Complex Microenvironments. Mater. Sci. Eng. C.

[75] Sun Han Chang R., Lee J.C.W., Pedron S., Harley B.A.C., Rogers S.A. (2019). Rheological Analysis of the Gelation Kinetics of an Enzyme Cross-Linked PEG Hydrogel. Biomacromolecules.

[76] Cordobés F., Partal P., Guerrero A. (2004). Rheology and Microstructure of Heat-Induced Egg Yolk Gels. Rheol. Acta.

[77] TA Instruments Gelation Kinetics from Rheological Experiments. https://www.tainstruments.com/pdf/literature/RH103.pdf.

[78] Ravanbakhsh H., Bao G., Latifi N., Mongeau L.G. (2019). Carbon Nanotube Composite Hydrogels for Vocal Fold Tissue Engineering: Biocompatibility, Rheology, and Porosity. Mater. Sci. Eng. C.

[79] Avallone P.R., Raccone E., Costanzo S., Delmonte M., Sarrica A., Pasquino R., Grizzuti N. (2021). Gelation Kinetics of Aqueous Gelatin Solutions in Isothermal Conditions via Rheological Tools. Food Hydrocoll..

[80] Cox T., Madsen C. (2017). Relative Stiffness Measurements of Cell-Embedded Hydrogels by Shear Rheology in Vitro. Bio-Protocol.

[81] Cox T.R., Erler J.T. (2011). Remodeling and Homeostasis of the Extracellular Matrix: Implications for Fibrotic Diseases and Cancer. Dis. Model. Mech..

[82] Dell A.C., Wagner G., Own J., Geibel J.P. (2022). 3D Bioprinting Using Hydrogels: Cell Inks and Tissue Engineering Applications. Pharmaceutics.

[83] Marques C.F., Diogo G.S., Pina S., Oliveira J.M., Silva T.H., Reis R.L. (2019). Collagen-Based Bioinks for Hard Tissue Engineering Applications: A Comprehensive Review. J. Mater. Sci. Mater. Med..

[84] Bachmann B., Spitz S., Schädl B., Teuschl A.H., Redl H., Nürnberger S., Ertl P. (2020). Stiffness Matters: Fine-Tuned Hydrogel Elasticity Alters Chondrogenic Redifferentiation. Front. Bioeng. Biotechnol..

[85] Ren Y., Zhang H., Wang Y., Du B., Yang J., Liu L., Zhang Q. (2021). Hyaluronic Acid Hydrogel with Adjustable Stiffness for Mesenchymal Stem Cell 3D Culture via Related Molecular Mechanisms to Maintain Stemness and Induce Cartilage Differentiation. ACS Appl. Bio Mater..

[86] Herrada-Manchón H. (2022). Formulación y Caracterización de Tintas Orgánicas Para (Bio)Impresión 3D. Ph.D. Thesis.

